# Influence of Dietary Supplementation with a Blend of Essential Oils, Tannins and Bioflavonoids on Milk Yield and Nutritional Properties and on Economic Sustainability of Buffalo Breeding

**DOI:** 10.3390/ani16111649

**Published:** 2026-05-28

**Authors:** Federica Dibennardo, Lorenzo Infascelli, Piera Iommelli, Raffaella Tudisco, Allegra Iasi, Nadia Musco, Federico Infascelli

**Affiliations:** 1 Department of Veterinary Medicine and Animal Production, University of Napoli Federico II, 80100 Naples, Italy; dibennardofederica@gmail.com (F.D.); tudisco@unina.it (R.T.); allegra.iasi9@gmail.com (A.I.); federico.infascelli@unina.it (F.I.); 2 Department of Economics and Law, University of Cassino and Southern Lazio, 03043 Cassino, Italy; lorenzo.infascelli@unicas.it; 3 Department of Experimental Medicine, University of Rome “Tor Vergata”, 00133 Rome, Italy; piera.iommelli@uniroma2.it

**Keywords:** buffalo breeding, nutritional supplement, economic sustainability, milk quality, milk fatty acids profile

## Abstract

The use of natural nutritional supplements has become increasingly common in livestock nutrition; nevertheless, because of its additional costs, a careful cost/benefit evaluation is essential to justify their practical application. The present study aimed to evaluate the effects of a blend of essential oils (EOs) included in the diet of dairy buffaloes on milk yield, chemical composition, milk urea nitrogen (MUN), and fatty acid profile. In addition, a detailed economic analysis was conducted to assess the overall profitability of this dietary strategy. A total of 98 buffaloes were enrolled in the trial and divided into two groups: a control group (C) and a treated group (T). The treated animals received 45 g/head/day of an essential oil blend in addition to a total mixed ration. An increase in milk yield was observed in the treated group at two sampling points, whereas no significant differences emerged in milk chemical composition. In contrast, MUN levels were consistently lower in the treated group throughout the trial. The fatty acid profile remained largely unchanged, except for higher levels of trans vaccenic acid (TVA) and eicosapentaenoic acid (EPA) in the treated animals. The economic analysis indicated that EO supplementation was financially advantageous for the farm. Overall, these findings suggest that essential oil blends may represent a promising nutritional strategy to improve production efficiency and milk quality in dairy buffalo. Future studies should investigate long-term effects, optimal dosages, and the mechanisms underlying the observed metabolic responses to support wider practical adoption.

## Simple Summary

Natural feed additives such as essential oils, tannins, and bioflavonoids are increasingly used in livestock nutrition to improve production efficiency and sustainability. This study evaluated the effects of a blend of these compounds on milk yield, composition, and economic performance in dairy buffaloes. The results showed increased milk yield, reduced milk urea nitrogen, and improved profitability, suggesting that this supplementation strategy can enhance both productivity and environmental sustainability in buffalo farming.

## 1. Introduction

In recent years, the dairy industry has faced increasing pressure to improve production efficiency and quality while adopting more sustainable practices [1]. According to Priyashantha [2], improving milk quality is crucial for achieving several Sustainable Development Goals (SDGs) outlined by the United Nations’ 17 in the 2030 Agenda (UN, 2015). Indeed, it contributes to SDG 3 (Good Health and Wellbeing) by providing essential nutrients and ensuring safe consumption and improving public health. Animal feeding strategies play a pivotal role in safeguarding natural ecosystems (SDG 15, Life on Land) and enhancing production efficiency and milk quality, primarily through the modulation of rumen fermentation. In the past, antimicrobial compounds were used to manipulate the rumen environment and improve feed conversion ratios [3,4,5,6]. However, the European Commission’s ban on these substances in animal nutrition [7], driven by concerns over antibiotic resistance, has catalysed the search for natural, bioactive alternatives [8,9,10,11]. Among these alternatives, plant-derived secondary metabolites, specifically essential oils (EOs), have emerged as promising candidates. EOs are aromatic, volatile, lipophilic compounds [12] which mainly work as antimicrobial, antioxidant and anti-inflammatory agents [13]. These compounds have been recognized as safe for use in human and animal foods [14,15]. Several in vitro and in vivo studies [16,17,18] have demonstrated the efficacy of essential oil supplements in selectively inhibiting methanogenic archaea and hyper-ammonia-producing bacteria. This reduces enteric methane emissions, improves nitrogen utilisation and increases digestion efficiency and milk yield. Nevertheless, results for milk yield and composition remain inconsistent. Elazab et al. [19] found no significant differences in milk yield or chemical composition when supplementing the diet of Holstein dairy cows with ginger or rosemary essential oils or a blend of both. As reported by the authors, the dosage of the bioactive compound present in the experimental EOs was considered for the purpose of obtaining results on methane production; however, this was not sufficient for the observation of effects on milk yields. In contrast, Daning et al. [20] reported that the addition of galangal EO to the diet of dairy cow significantly increased milk polyunsaturated fatty acids without affecting milk yield. Such discrepancies must be attributable to differences in essential oil class, dosage, and duration of administration [21,22]. While EOs can enhance milk production by improving feed and nutrient utilisation and optimise rumen fermentation, their effects are not always positive. Depending on the specific EO and dosage used, they may disrupt rumen microbial balance, reducing VFA production and milk yield, or decrease feed intake due to palatability issues, ultimately compromising animal performance.

Recent research has increasingly focused on blends of multiple essential oils which may offer superior benefits through synergistic effects [23,24]. The presence of several bioactive compounds allows us to target various microbial pathways simultaneously, decreasing the risk of rumen microbiota adaptation [21], and by allowing lower inclusion levels of individual compounds, potentially avoids palatability issues or side effects [25]. Bach et al. [26] observed a decrease in methane production with no effects on milk performance in dairy cows fed a diet supplemented with a blend of essential oils, whereas Rossi et al. [27] reported a significant increase in milk yield without affecting the chemical composition of the milk by adding a blend of essential oils, mainly from cloves, coriander seeds and geranium, and of tannins and bioflavonoids to the dairy cows’ diet. However, whether such dietary strategies can be effectively applied to other dairy species remains less explored. The use of EOs in the dairy buffalo diet is very limited [28] and blends of EOs have not been tested yet. The interest in dairy buffalo (*Bubalus bubalis*) is related to its great economic importance in southern Italy, primarily for producing mozzarella cheese [29,30,31] and to a lesser extent for supplying buffalo meat [32]. Buffalo are more efficient than dairy cows at digesting structural carbohydrates [33,34] and protein [35] from feed, which leads to better overall feed efficiency and improved nitrogen retention owing to a more efficient incorporation of ammonia nitrogen into microbial protein by rumen bacteria [11,36,37]. However, most dairy buffalo farms use an intensive breeding system characterized by a high proportion of concentrates in the diet. In turn, this results in the emergence of several technopathies involving rumen fermentation [38], which have detrimental effects on the nutritional quality of milk. Therefore, improving nutritional management represents a strategy for averting the aforementioned disorders. The supplementation of the diet with modulators of rumen fermentation has been demonstrated to be useful for this purpose. In light of the aforementioned results in dairy cows and to address the gap regarding the buffalo species, the hypothesis of this study was that adding a blend of essential oils, tannins and bioflavonoids to the diet of lactating buffalo cows would positively influence rumen environment and milk productive performance. Consequently, this trial was carried out at an intensive buffalo farm with the aim to evaluate the impact of incorporating such a blend into the diet on milk yield, chemical composition, and fatty acid profile.

## 2. Materials and Methods

The trial was conducted in 2024 for five months at a commercial buffalo farm in the province of Caserta, Campania Region, Italy (103 m a.s.l., 41°11′ N 14°03′ E, with an average rainfall of 906 mm and a mean temperature of 3.0–31.0 °C). It was approved by the Ethical Animal Care and Use Committee of the University of Naples Federico II (protocol number 2019/0013729, dated 8 February 2019).

### 2.1. Animals and Diets

Ninety-eight Italian Mediterranean buffalo cows were equally divided into two groups (C, control vs. T, treated) homogeneous in terms of parity (3.33 ± 0.4 vs. 3.41 ± 0.5; *p* > 0.05), body weight (622 ± 24 kg vs. 625 ± 21 kg; *p* > 0.05), days in milk (29.9 ± 6 vs. 30.0 ± 5; *p* > 0.05) and previous milk yield (2350 ± 133 kg vs. 2240 ± 128 kg; *p* > 0.05). Animals were individually fed and allocated to individual open yards with a permanent bedding area (8 m^2^/head), an exercise area (12 m^2^/head) and a feeding area (3.5 m^2^/head), with free access to water. The animals were fed a total mixed ration (TMR), the ingredients of which are reported in Table 1. The diet of group T was supplemented with 45 g/head/day of Anavrin (Vetos Europe SAGL, Via delle Industrie 18, 6593 Cadenazzo, Switzerland), a coated blend of EOs, mainly from cloves (*Syzygium aromaticum*) and geraniums (*Pelargonium cucullatum*), tannins from chestnuts (*Castanea sativa*), and bioflavonoids from olives (*Olea europaea*). The relative concentrations of the active principles in the product were EO:CT:BF = 1:2.5:0.1. The supplement was top-dressed onto the TMR. TMR refusals were collected daily to calculate the individual dry matter intake (DMI). The animals’ body condition score (BCS) was evaluated monthly by the same operators using a 1–5 scale. where 1 = emaciated, 2 = thin, 3 = average, 4 = fat and 5 = obese [39].

### 2.2. Feed Analysis

Before feeding, samples of total mixed ration (TMR) were collected weekly from the feed fence, oven-dried at 65 °C, milled through a 1 mm screen and analysed for dry matter (DM), crude protein (CP), ether extract (EE) and ash content, as suggested by AOAC [40]. The ID numbers were 2001.12. 978.04, 920.39 and 978.10, 930.05, respectively. Structural carbohydrates were determined according to Van Soest et al. [41], while starch was assessed using the polarimetric method (Polax L. Atago, Tokyo, Japan) in accordance with the official procedure [40]. The TMR’s nutritive value (UFL = 1700 kcal of net energy for lactation) was calculated as reported by INRA [42]. The fatty acid (FA) profile of the TMRs was determined by extracting approximately 200 mg of fat as described by Folch et al. [43], subsequently methylating it according to Christie [44], and injecting it into a gas chromatograph (GC) with a flame ionisation detector (GC-FID; TRACE 1310 system equipped with an AS 1310 autosampler and an AI 1310 autoinjector; Thermo Fisher Scientific, Milan, Italy). A temperature program of 100 °C for 5 min, 100–240 °C at 4 °C/min and a final isotherm at 240 °C for 20 min was used with the Omegawax 250 capillary polar column (Supelco, Bellefonte, PA, USA), 30 m × 0.25 mm, 0.25 μm (L × I.D., film thickness). The injector and detector temperatures were both set to 250 °C, the injection volume was set to 0.5 µL and the split ratio was set to 1:50. The carrier gas (He) had a flow rate of 1 mL/min, the make-up gas (N_2_) flow was 40 mL/min, the H_2_ flow was 35 mL/min and the air flow was 350 mL/min. The data were processed using Chromeleon™ Data System software (version 7.2.9) (Thermo Fisher Scientific, Milan, Italy), and the individual fatty acid methyl esters (FAMEs) were identified by comparing the peak retention times with Supelco standards (Merck KGaA, Darmstadt, Germany). The concentrations of individual FAs were expressed as g/100 g with 100 g representing the sum of all areas of the identified FAMEs. All analyses were performed in triplicate.

### 2.3. Milk Analysis

Individual milk yield (MY) was recorded daily using Delpro software (version 5.2.1), while individual milk samples (from the two daily milkings) were collected monthly, for a total of 5 samplings, and analysed for chemical composition (fat, protein, lactose) and urea by using Milko Scan FT 6000 (Foss Matic, Hillerod, Denmark) calibrated for buffalo milk. To study the fatty acid profile of the milk, total fat was extracted using a mixture of hexane, propanol and isopropanol (3:2:1, *v/v*) [45], and trans-methylated according to the Christie [40] procedure, as modified by Chouinard et al. [46]. The methyl esters were analysed using a ThermoQuest 8000TOP gas chromatograph (Thermo Electron Corporation, Rodano, Milan, Italy) equipped with a flame ionisation detector and a CP-SIL 88 fused silica capillary column (100 m × 0.25 mm internal diameter with a film thickness of 0.2 µm; Varian Inc., Walnut Creek, CA, USA). The temperature ramp was set to 70 °C for 4 min, then increased at a rate of 13 °C/min to 175 °C for 27 min, then increased at a rate of 3 °C/min to 215 °C for 38 min, then decreased at a rate of 10 °C/min to 70 °C. The injector and detector temperatures were 250 °C and 260 °C, respectively. The gas flows were as follows: carrier gas (helium): 1 mL/min; hydrogen: 30 mL/min; air: 350 mL/min; make-up gas (helium): 45 mL/min. The fatty acid peaks were identified by comparing them with a standard mixture of fatty acid methyl esters (Larodan Fine Chemicals AB, Limhamnsgårdens, Malmö, Sweden). The identification of the isomers of conjugated linoleic acid (CLA) was determined by comparing the sample chromatograms with those of the individual purified isomers (CLA cis-9 trans-11; CLA trans-10 cis-12;). (Larodan Fine Chemicals AB, Limhamnsgårdens, Malmö, Sweden). All analyses were performed in triplicate.

Finally, the mozzarella cheese yield (MCY) was estimated by the formula of Altiero et al. [47]:

MCY (%) = [3.5 (% protein) + 1.23 (% fat) − 0.88]/100

### 2.4. Statistical Analysis

Data were analysed using a two-way ANOVA for repeated measure according to the following model:

yijk = μ + Gi + Sj + GxSij + d(Gi) + eijk

where yijk = single observation; μ = general mean; Gi = group effect (i = C and T); Sj = sampling effect (j = I, II, … V); GxS = interaction between group and sampling effect; d (Gi) = random effect of animal nested within group; eijk = experimental error.

Tukey’s test was used to compare the mean values, and the differences were considered significant at *p* < 0.05. All the analyses were performed using JMP^®^ (version 14; SAS Institute, Cary, NC, USA) software.

## 3. Results and Discussions

In Table 2 the TMR chemical composition, nutritive value and fatty acid profile are reported. The protein content of the TMR was around 140 g/kg DM, which is a level commonly adopted on intensive buffalo farms in Italy. In contrast, the starch concentration was 266.3 g/kg DM, higher than those typically used (220–230 g/kg DM) on most buffalo farms, remaining nevertheless within the range suggested for dairy buffaloes [48].

TMR protein and energy concentrations met the respective requirements for lactating buffalo cows [49]. Among the fatty acid classes, the PUFA showed the highest concentrations (and of particular interest appears the high concentration of the omega 3 fatty acids which, according to Arvidsson et al. [50], is probably due to the presence of grass silage among the TMR ingredients).

**Figure 1:**
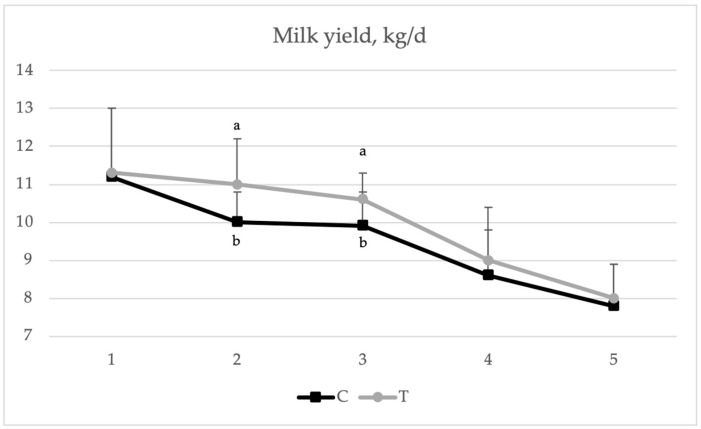
Milk yield comparison between groups (mean ± standard deviation). C: control group; T: treated group. Different letters indicate statistically significant differences at *p* < 0.05.

No TMR refusals were detected for both the groups. As presented in Table 3 and Table 4, no significant differences were found in body condition score, dry matter intake, average milk yield, milk fat, protein and lactose between the control and treated groups. The values of BCS were similar to those reported in previous research performed in the same area [51] with similar diet, and the DMI was consistent with that typically observed for medium-to-high-producing buffalo cows and was unaffected by treatment, confirming the good palatability of the experimental products. Indeed, the buffalo species is known to be wary of the introduction of new feeds in their diet [52]. Neither BCS nor DMI were significantly affected by the month of lactation. Although a slight decrease of DMI in the first 50 days of lactation is reported also for buffalo cows, their low catabolic activity means that any unmet nutritional requirements tend to results in a decrease in the milk yield rather than mobilization of body reserve [53]. With regard to milk yield, analysis of the data collected over the course of the trial suggests a beneficial effect of supplementation of the EO blend. In fact, as depicted in Figure 1, a statistically significant increase in milk yield was observed in the treated group beginning at the second and third sampling points.

**Figure 2:**
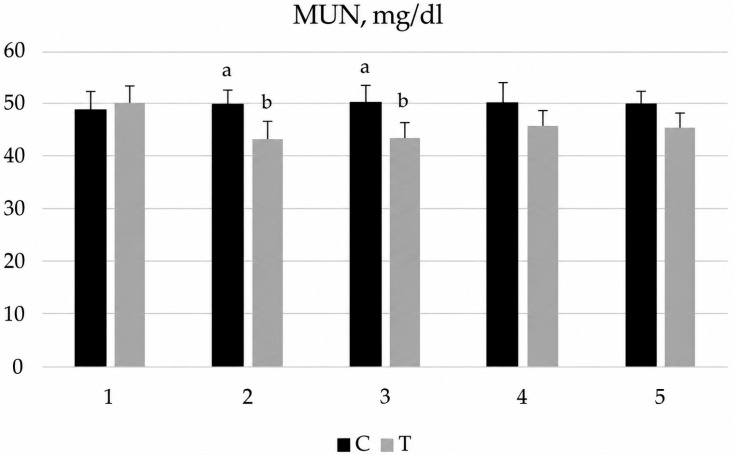
Milk urea (MUN) comparison between groups (mean ± standard deviation). C: control group; T: treated group. Different letters indicate statistically significant differences at *p* < 0.05.

MUN levels in the treated group were significantly (*p* < 0.05) lower than those of control group during the entire trial, with the exception of the first sampling (Figure 2). Firstly, it should be noted that maximum tolerable levels of MUN (36–42 mg/100 mL), considered to be risky for dairy cows, are easily reached and exceeded in buffalo cows [52]. As proposed by Peyraud et al. [53], the underlying factors contributing to elevated or reduced urea values include excess or deficiency of fermentable energy, as well as protein concentration and degradability. In the present trial, both groups were fed the same diet, thus excluding the possibility that the observed difference in MUN could be attributed to the dietary factors. Instead, the improved nitrogen use observed in the treated group could be attributed to the modulating activity of the EO blend on rumen fermentation, as previously suggested by Braun et al. [54]. According to Ugbogu et al. [55], EOs are responsible for increases of the duodenal flow of rumen undegraded protein by reducing the conversion of protein into NH_3_. In addition, a reduction in MUN has been also reported by Giannenas et al. [56], who supplemented the diet of lactating ewes with a blend of EOs, whereas Benchaar et al. [57] found that in lactating cows supplemented with ionophore antibiotic, the MUN values were successively normalized by using EOs. MUN and urinary urea elimination above a certain blood content threshold [58] are strictly linked; consequently, a significant loss of nitrogen and energy occurs in cases of increased MUN levels. According to Caprarulo et al. [59], even if the nitrogen excreted by livestock is necessary to guarantee soil fertility and to sustain the agricultural circularity, its excesses could determine soil and water degradation [60,61] as high levels of reactive nitrogen are critical in soil and water acidification, eutrophication and loss of biodiversity across both terrestrial and aquatic ecosystems [62,63,64]. Nutritional interventions, particularly the use of feed additives, are among the most actively developed approaches for reducing emissions in ruminants [65,66] and thus for realizing a higher safeguarding of natural ecosystems.

The effect of supplementing the diet with the essential oil blend on the fatty acid profile of milk, which helps to indicate the health properties of food [67], is reported in Table 5 and Table 6 (classes and single fatty acids, respectively). No significant differences were detected in the total amount of saturated, monounsaturated or polyunsaturated fatty acids, nor in omega-6 PUFAs, omega-3 PUFAs or CLA. Consequently, none of the ratios were affected by the treatment. However, since buffalo milk is mainly consumed as cheese, small differences in milk composition could be exacerbated in cheese products. On the contrary, significant differences in the levels of single fatty acids emerged between groups for trans vaccenic acid (*p* < 0.05) and eicosapentaenoic acid (*p* < 0.01), with both values being higher in the treated group. TVA is ubiquitous in fats derived from ruminants and in human dairy products such as milk and butter [68]. Notably, TVA is also the predominant trans fatty acid in human milk. This FA has been demonstrated to possess important biological activities as demonstrated by Degen et al. [69], whose findings suggest that supplementing with milk lipids containing TVA generates a remarkable cytotoxic effect in HT29 cancerogenic cells due to conversion to conjugated linoleic acid (CLA). In addition, the direct anti-carcinogenic effect of TVA has been demonstrated by Lim et al. [70], who reported that TVA inhibited the proliferation of MCF-7 human breast adenocarcinoma cells by down-regulating the expression of Bcl-2 and procaspase-9. Omega-3 PUFAs, mainly docosahexaenoic acid (DHA) and eicosapentaenoic acid (EPA), are globally known as important for human health due to their protective activity to cardiovascular disease [71,72]. Omega-3 PUFAs have been shown to improve cardiac function [73], decrease blood triacylglycerol concentration [74], lower blood pressure [73], reduce depression [75] and enhance cognitive function [76]. More recently, a positive effect of EPA on the immune system and on microbiome composition has also been reported [77].

Several natural feed supplements, especially those containing tannins and specific classes of fatty acids, are able to improve milk FA profile, boosting PUFAs, n-3 [78] and DHA [79,80]. Nevertheless, different dosages and mechanisms of action are involved, since the supplementation employed in those works was used at higher dosages and was characterized by a specific lipidic profile that passes into the milk. EOs presumably have worked on rumen microflora, changing metabolic pathways.

Therefore, the significant increase in the levels of TVA and EPA in the milk of buffalo cows fed the diet supplemented with a blend of essential oils should improve the nutritional characteristics of the milk.

## 4. Economic Analysis

Economic analysis has been made just taking into account that the cost of the EOS blend was EUR 3.50/kg and the milk selling price was EUR 1.70/kg. The dietary supplementation should also be advantageous from an economic point of view; in fact, analyzing Table 7, where the income and cost along the trial are reported, the additional income for group T compared to group C was around EUR 5100.

Considering the entire production chain, further increase in income could come from the sale of mozzarella. In fact, using Altiero et al.’s [47] formula, the mozzarella cheese yield (MCY) was estimated at 25.5% for both groups due to the lack of difference in average milk fat and protein levels. However, Zicarelli et al. [81] suggest that high levels of milk urea could affect MCY, with around 1% reduction in yield for every 10 mg/dL increase in MUN [82,83]). Therefore, due to the significantly higher level of MUN registered for group C, its MCY should be only 25%. Therefore, the amount of mozzarella produced by the treated group should be 1286.25 kg more than group C (Table 8). However, this estimation needs to be confirmed by further research measuring effective MCY.

## 5. Conclusions

The findings of this study indicate that dietary supplementation with a blend of EOs can represent a valuable supplementation in dairy buffalo farming. Increases in milk yield and reduction in MUN suggest a potential improvement in nitrogen utilisation efficiency, which is particularly relevant for both productive performance and environmental sustainability. Moreover, the increase in selected beneficial fatty acids, such as TVA and EPA, may contribute positively to the nutritional quality of buffalo milk. In addition, the economic evaluation demonstrated that the inclusion of essential oils in the diet was financially advantageous under the conditions of the present study, supporting the practical applicability of this approach in commercial farms. These findings highlight the potential of natural feed additives to enhance productivity while maintaining economic sustainability. These results are related to specific farm condition such as specific diet, animal genetics and environment; therefore, further research is required to investigate their interactions with different diet composition and management conditions to help to define more precise nutritional strategies. Overall, essential oil supplementation appears to be a promising tool for improving efficiency, product quality, and sustainability in dairy buffalo production systems.

## Figures and Tables

**Table 1:** TMR ingredients (kg as fed).

Sorghum silage	10.0
Grass silage *	8.0
Alfalfa hay	3.0
Polyphyte hay **	1.5
Corn mash	5.0
Wheat meal	2.0
Wheat distillers	1.0
Commercial concentrate ***	3.1
Vit-min mix	0.3

* *Lolium italicum* L.; ** *Avena sativa* L., *Lolium italicum* L., *Trifolium pratense* L. *** Soybean meal s.e., corn meal, dried beet pulp, CaCO3, NaCl, MgO, sodium bicarbonate, Vit A 60.000 IU, Vit D3 8000 IU, Vit E 90.00 mg, Iodium 4.00 mg, MnO 200.00 mg, Se 0.60 mg, Zn 200.00 mg.

**Table 2:** TMR chemical composition (g/kg DM), nutritive value (UFL/kg DM), and fatty acid profile (g/100 g of fat).

CP	139.7 ± 4.0
EE	38.2 ± 1.2
CF	235.4 ± 5.4
NDF	398.5 ± 1.1
ADF	204.3 ± 7.2
ADL	52.1 ± 1.3
Ashes	76.4 ± 2.0
Starch	266.3 ± 8.4
UFL/kg DM	0.91
∑ SFA	22.7 ± 1.1
∑ MUFA	20.3 ± 2.0
∑ PUFA	57.0 ± 3.2
ω6 PUFA	30.6 ± 1.4
ω3 PUFA	26.4 ± 1.7

CP: crude protein; EE: ether extract; CF: crude fibre; NDF: neutral detergent fibre; ADF: acid detergent fibre; ADL: acid detergent lignin; UFL: feed unit for lactation; SFA: saturated fatty acids; MUFA: monounsaturated fatty acid; PUFA: polyunsaturated fatty acid.

**Table 3:** Mean value of body condition score (BCS), dry matter intake (DMI, kg) and milk yield (MY, kg/h/d).

	BCS		DMI		MY	
Sampling Points	C	T	C	T	C	T
	3.2	3.3	17.1	17.3	9.5	10.0
1	3.1	3.2	17.0	17.1	11.2	11.3
2	3.2	3.2	17.0	17.1	10.0 b	11.0 a
3	3.2	3.3	17.1	17.3	9.9 b	10.6 a
4	3.2	3.3	17.1	17.4	8.6	9.0
5	3.3	3.3	17.1	17.4	7.8	8.0
p G	NS		NS		NS	
p S	NS		NS		*	
p G x S	NS		NS		*	
RMSE	0.08		0.18		2.24	

C: control group; T: treated group; RMSE: root mean square error; NS: not significant; * *p* < 0.05. Different letters indicate statistically significant differences at *p* < 0.05.

**Table 4:** Mean values of milk composition (%) and milk urea (MUN, mg/dL).

	Fat		Protein		Lactose		MUN	
Sampling Points	C	T	C	T	C	T	C	T
	8.1	8.1	4.7	4.7	5.1	5.0	49.7 a	45.6 b
1	7.5	7.4	4.7	4.6	4.9	5.0	48.7	49.5
2	7.8	7.5	4.5	4.7	5.3	5.1	49.6 a	43.2 b
3	8.0	8.0	4.7	4.6	5.1	5.1	50.3 a	43.4 b
4	8.6	8.8	4.8	4.9	5.0	5.0	50.0 a	45.9 b
5	8.8	9.1	4.7	4.8	5.0	5.0	50.0 a	45.6 b
p G	NS		NS		NS		*	
p S	**		**		**		*	
p G x S	NS		NS		NS		*	
RMSE	1.11		0.323		2.81		9.11	

C: control group; T: treated group; RMSE: root mean square error; NS: not significant; * *p* < 0.05; ** *p* < 0.01. Along the column and row, different letters indicate statistically significant differences at *p* < 0.05.

**Table 5:** Classes of milk fatty acid profile (% total fatty acids).

Group	C	T	p G	p S	p G x S	RMSE
SFA	72.2	68.7	NS	NS	NS	11.64
MUFA	24.8	22.44	NS	NS	NS	5.51
PUFA	2.49	2.47	NS	NS	NS	0.317
ω6	1.91	1.75	NS	NS	NS	0.236
ω3	0.159	0.159	NS	NS	NS	0.04
CLA	0.412	0.363	NS	NS	NS	0.106
PUFA/SFA	0.034	0.031	NS	NS	NS	0.005
ω6/ω3	12.35	11.78	NS	NS	NS	2.79
LA/ALA	16.9	16.13	NS	NS	NS	4.7
AA/EPA	2.81	2.27	NS	*	NS	0.75

C: control group; T: treated group; RMSE: root mean square error; NS: not significant; * *p* < 0.05.

**Table 6:** Single milk fatty acid profile (% total fatty acids).

	C	T	p G	p S	p G x S	RMSE
C4:0	3.610	3.905	NS	NS	NS	0.904
C6:0	1.483	1.545	NS	NS	NS	0.229
C8:0	1.267	1.035	NS	NS	NS	0.559
C10:0	2.976	2.384	NS	NS	NS	1.366
C11:0	0.090	0.079	NS	NS	NS	0.848
C12:0	3.119	3.012	NS	NS	NS	0.956
C13:0	0.085	0.082	NS	NS	NS	0.049
C14:0	12.71	12.84	NS	NS	NS	1.423
C14:1	0.318	0.353	NS	NS	NS	0.136
C15:0	0.828	0.860	NS	NS	NS	0.254
C16:0	35.91	35.95	NS	NS	NS	2.720
C16:1	1.997	2.02	NS	NS	NS	0.737
C17:0	0.455	0.315	NS	NS	NS	0.182
C18:1 *cis* 6	0.131	0.073	NS	NS	NS	0.079
C18:0	9.58	10.27	NS	NS	NS	1.510
C18:1 *trans* 9	0.216	0.338	NS	NS	NS	0.180
C18:1 *trans* 11 ω7 (TVA)	1.316 b	1.605 a	*	NS	NS	0.352
C18:1 cis 9 ω9	20.38	19.94	NS	NS	NS	1.779
C18:1 *cis* 11 ω7	0.143	0.177	NS	NS	NS	0.073
C18:1 *cis* 12	0.049	0.046	NS	NS	NS	0.031
C18:2 *trans* ω6	0.036	0.040	NS	NS	NS	0.047
C18:2 *cis* ω6 (LA)	1.727	1.607	NS	NS	NS	0.212
C20:0	0.131	0.123	NS	NS	NS	0.04
C18:3 ω3 (ALA)	0.104	0.121	NS	NS	NS	0.035
CLA1	0.150	0.126	NS	NS	NS	0.087
CLA2	0.194	0.202	NS	NS	NS	0.080
C20:2 ω6	0.0228	0.023	NS	NS	NS	0.0066
C22:0	0.076	0.063	NS	NS	NS	0.030
C20:3 ω6	0.019	0.018	NS	NS	NS	0.0075
C22:1	0.030	0.036	NS	NS	NS	0.018
C20:4 ω6 (AA)	0.079	0.077	NS	NS	NS	0.020
C24:0	0.035	0.024	NS	NS	NS	0.019
C20:5 ω3 (EPA)	0.030 B	0.042 A	**	*	**	0.0094
C24:1	0.023	0.019	NS	NS	NS	0.007
C22:4 ω6	0.018	0.018	NS	NS	NS	0.008
C22:5 ω3 (DPA)	0.018	0.015	NS	NS	NS	0.0051

C: control group; T: treated group; RMSE: root mean square error; * *p* < 0.05; ** *p* < 0.01. Along the row different letters indicate a significant differences *p* < 0.01.

**Table 7:** Income by milk yield during the experimental trial (150 days).

Group	C (49 Heads)	T (49 Heads)	Δ C vs. T
Milk yield (kg)	69,825	73,500	3675
Income milk (1.7 EUR/kg)	118,702,50	124,950,00	6247,50
Cost EO blend (EUR)	-	1,157,625	1,157,625
Final income (EUR)	118,702,50	123,792,375	5,089,875

**Table 8:** Estimated kg of mozzarella cheese produced along the trial.

Group	C	T	Δ C vs. T
Milk yield (kg)	69,825	73,500	3675
MUN (mg/dL)	49.7	45.6	4.1
Mozzarella cheese yield (%)	25.0	25.5	0.5
Mozzarella cheese (kg)	17,456.25	18,742.50	1286.25

## Data Availability

The data presented in this study are available on reasonable request from the corresponding author.
